# Genome-Wide Analysis of BPC Gene Family in Soybean and the Role of *GmBPC8* in Floral Organ Development in *Arabidopsis*

**DOI:** 10.3390/plants15192940

**Published:** 2026-09-26

**Authors:** Kuo Yang, Binbin Li, Ruihong Sun, Yanfu Pan, Shangjing Guo, Chongyang Li

**Affiliations:** 1 College of Agriculture and Biology, Liaocheng University, Liaocheng 252000, China; yangkuo@lcu.edu.cn (K.Y.); l2019in@126.com (B.L.); srh0115@163.com (R.S.); panyanfu2006@163.com (Y.P.); 2 College of Life Sciences, Qingdao Agricultural University, Qingdao 266109, China

**Keywords:** GmBPC8, soybean, expression patterns, floral development, gene family

## Abstract

The BARLEY B RECOMBINANT/BASIC PENTACYSTEINE (BBR/BPC) transcription factors are widely involved in plant development and abiotic stress responses. In this study, ten *GmBPC* genes were identified in the soybean genome, and their characteristics and expression patterns were comprehensively analyzed. Based on phylogenetic relationships, the *GmBPCs* were classified into two groups, and members within the same group exhibited high similarity in motif compositions and gene structures. Expression profiling indicated that *GmBPCs* were expressed in all tissues, with most showing high transcript levels in seeds. The *GmBPCs* exhibited different expression profiles in soybean under drought and heat stresses. Ectopic expression of *GmBPC8* in *Arabidopsis* led to abnormal floral development and reduced the seed setting rate. Additionally, GmBPC8 could form both homodimers and heterodimers with other GmBPC proteins. Collectively, these findings provide a foundation for elucidating the functional roles of BPC transcription factors.

## 1. Introduction

Normal floral organ development is a prerequisite for sexual reproduction and seed set in flowering plants. Flower development is coordinately regulated by both external environmental factors and internal physiological signals, representing a highly ordered and tightly controlled biological process [1]. Within this regulatory network, transcription factors (TFs) precisely orchestrate multiple aspects of floral development by activating or repressing the expression of target genes [2]. Among these, the MADS-box and AP2/ERF TFs serve as the core determinants of floral organ identity, as systematically illustrated by the classical ABCDE model [2]. In addition, other TFs, such as MYB, bHLH, and BPC, are involved in multiple processes of flower development [3,4,5]. Abnormalities in floral organ development often reduced fertility in plants [6,7,8].

At present, a large number of TF families have been identified in plants. Compared with other families, the BBR/BPC TF family has fewer members in plants [9]. BPC proteins are poorly characterized TFs with an unusual arrangement of five conserved cysteine residues in the C-terminus, which is required for DNA binding, whereas the N-terminus is more divergent [9]. BPC can directly bind to GA-rich sequences of target gene promoters and regulate their expression [9,10]. BPC proteins are involved in plant growth and development [11,12,13,14]. *Arabidopsis BPC1* is expressed in all floral organs, especially in the ovule [15]. In *Arabidopsis thaliana*, the *BPC* genes exhibit a high degree of functional redundancy, as neither single mutants nor double *bpc* mutants result in abnormal plant phenotypes. However, the triple mutant *bpc123* and quadruple mutant *bpc1-1 bpc2 bpc4 bpc6* cause apparent reproductive organ defects [16,17]. The *bpc123* triple mutants show short siliques and reduced seed set [18]. The *bpc1-1 bpc2 bpc4 bpc6* quadruple mutant exhibits compact inflorescences, persistent floral bud closure, aberrant carpel and ovule development, and largely reduced fertility [16]. Moreover, BPC proteins directly regulate key downstream target genes, including *SPATULA* (*SPT*) and *NO TRANSMITTING TRACT* (*NTT*), to control gynoecium and septum development, both of which are critical for carpel and transmitting tract formation [18].

In addition, BPC TFs participate in multiple abiotic stress and phytohormone responses [19]. The *bpc* quadruple mutant displayed a markedly impaired ethylene response, with longer and thinner hypocotyls [16]. The *Arabidopsis bpc* multiple mutant display lower sensitivity to cytokinin [20]. The *Arabidopsis* BPC proteins could directly bind to the cytokinin-responsive genes *ARABIDOPSIS RESPONSE REGULATOR 7* (*ARR7*), *SHOOTMERISTEMLESS* (*STM*), and *BREVIPEDICELLUS/KNAT1* (*BP*) [20,21,22,23]. In *Arabidopsis thaliana*, BPC1/BPC2 enhance salt tolerance by directly repressing *GALACTAN SYNTHASE 1* (*GALS1*) expression, and also improve waterlogging tolerance by positively regulating the expression of the peroxidase gene *Prx28* [24,25]. Soybean [*Glycine max* (L.) Merr.] is a globally important crop that provides valuable edible oils and proteins and plays a critical role in the world economy [26,27]. Currently, the *BPC* gene family has been studied in multiple plants, including *Arabidopsis thaliana*, apple, coconut palm, cucumber, maize, rice, and tomato [14,28,29,30,31,32]. However, systematic analysis of the BPC family in soybean remains poorly understood. Here, ten *GmBPC* genes were identified in the soybean genome. We systematically analyzed the phylogenetic relationships, conserved domains, and *cis*-regulatory elements of *GmBPCs*. The tissue-specific and abiotic stress profiles of *GmBPCs* were examined. Based on the distinct tissue-specific expression pattern, we selected *GmBPC8* for functional characterization by ectopic overexpression in *Arabidopsis* to determine the role of *GmBPC8* in floral organ development and seed set. Furthermore, yeast two-hybrid (Y2H) assays revealed that GmBPC8 interacts with other GmBPC proteins. Collectively, this study provides new insights into *GmBPC* functions in soybean and uncovers a role for *GmBPC8* in reproductive development.

## 2. Results

### 2.1. Identification and Characterization of GmBPCs

BLASTP and HMMER searches were performed to identify BPC family members in the soybean genome. In this study, ten *GmBPCs* were identified (Table 1). Soybean *GmBPCs* were named *GmBPC1*–*GmBPC10* based on their chromosomal positions (Figure S1). The gene length of *GmBPCs* varied from 1345 bp (*GmBPC6*) to 4141 bp (*GmBPC4*), and the protein length ranged from 279 aa (GmBPC2 and GmBPC8) to 338 aa (GmBPC1). The molecular weight varied from 30,943.53 Da (*GmBPC2*) to 37,455.40 Da (*GmBPC4*). The GRAVY and theoretical pI values of the *GmBPC* genes are listed in Table 1. All the *GmBPC* genes mapped on chromosomes Chr04–Chr09. Among them, Chr05, Chr07, Chr08, and Chr09 each contained two *GmBPC* genes, whereas Chr04 and Chr06 each carried only one (Table 1).

### 2.2. Phylogenetic and Synteny Analysis

The phylogenetic tree was constructed using the neighbor-joining (NJ) method based on 42 proteins from seven plant species. According to phylogenetic analysis, these BPC proteins were divided into two groups, with 21 in Group I and 21 in Group II (Figure 1). In addition, *GmBPC1*, *GmBPC3*, *GmBPC4*, and *GmBPC7* were in group I, and the remaining *GmBPCs* were in Group II. Gene duplication is vital for the emergence of new gene functions and the expansion of gene families [33]. Among the ten *GmBPC* genes, three pairs of segmental duplications were identified in the soybean genome, such as *GmBPC1*/*GmBPC4*, *GmBPC3*/*GmBPC7*, and *GmBPC5*/*GmBPC9* (Figure 2). These findings imply that segmental duplication may affect the expansion of the soybean BPC family.

### 2.3. Gene Structure and Protein Sequence Analysis of GmBPCs

To further investigate the characteristics of *GmBPCs*, we analyzed their gene structures. The results showed that *GmBPCs* within the same phylogenetic group shared similar exon–intron structures (Figure 3). The number of introns in all *GmBPC* genes varied from 0 to 3. In addition, the number and spatial arrangement of exons also differed markedly between the two groups. Furthermore, ten conserved motifs were analyzed using the MEME tool (Figure 3). All the *GmBPC* genes possessed six common motifs, including motif 1, motif 2, motif 3, motif 6, motif 7, and motif 8, whereas motif 4 was specific to group I, and motif 10 was unique to group II. These results suggest that *GmBPC* genes have similar motifs and gene structure composition in the same evolutionary group. Previous studies have shown that BPC proteins are highly conserved in plant species [31,34]. In soybean, all the GmBPC proteins contained conserved Zn5C, DNA-binding, and WAR/KHGTN at the C-terminus (Figure S2). Moreover, Zn5C and DNA-binding motifs corresponded to motif 1, and WAR/KHGTN motif corresponded to motif 2.

### 2.4. Promoter Analysis of GmBPC Genes

Promoter analysis of *GmBPCs* was carried out using the PlantCARE website. Nineteen *cis*-acting elements were classified into three major categories, including hormones, growth and development, stresses (Figure 4). The hormone-responsive elements contained those responsive to abscisic acid, auxin, gibberellin, methyl jasmonate, and salicylic acid. All *GmBPC* genes contained 1–3 types of hormone-responsive elements, except *GmBPC6*. The growth- and development-related elements of *GmBPCs* included those involved in circadian control, flavonoid biosynthesis, meristem expression, and palisade mesophyll cells. More than half of the *GmBPC* genes contained 1–3 types of growth- and development-related elements. The stress-responsive elements were associated with low-temperature, drought, defense and stress, wound, and anaerobic induction. All *GmBPCs* contained 2–4 types of stress-related elements.

### 2.5. Expression Profiles of GmBPCs in Different Tissues

Exploring gene expression patterns is critical for elucidating gene functions. We analyzed the expression profile of *GmBPCs* in nine different tissues, including root, root hair, nodule, stem, leaf, shoot apical meristem (SAM), pod, seed, and flower, based on transcriptome data from the Phytozome database. As shown in Figure 5, most *GmBPCs* were expressed in all tissues, except for *GmBPC3* and *GmBPC7*. The expression levels of *GmBPC1* and *GmBPC10* were relatively high across all tissues (Figure 5). Some *GmBPCs* exhibited tissue-specific expression, indicating functional diversification during development. For instance, *GmBPC2* and *GmBPC9* showed the highest expression in roots and SAM, respectively, whereas all other *GmBPCs* were predominantly expressed in seeds. Interestingly, the *GmBPC* genes had similar expression patterns in the same group of the evolutionary tree.

### 2.6. Expression Responses of GmBPCs Under Abiotic Stresses

To investigate the potential roles of *GmBPCs* in response to abiotic stresses, we measured the expression patterns under drought and heat stresses. The expression levels of *GmBPCs* gradually increased and reached a maximum from 2 to 4-fold change at 12 h or 24 h under PEG treatment (Figure 6). Similarly, most *GmBPCs* were gradually increased at 24 h when exposed to heat stress, except for *GmBPC6*. There was no prominent difference in the expression level of *GmBPC6* under heat stress. Thus, *GmBPCs* may be involved in different stresses.

### 2.7. Overexpression of GmBPC8 Reduces Seed Set in Arabidopsis thaliana

Given the distinct tissue-specific expression pattern of *GmBPC8*, we further cloned this gene and constructed its overexpression vector. We generated multiple independent *GmBPC8*-overexpressing *Arabidopsis* lines and selected three lines (OE1–OE3) with relatively high expression levels by qRT-PCR for further characterization (Figures S3 and S4). No significant difference in plant height was observed between the wild type (WT) and transgenic lines (Figure S5). However, *GmBPC8*-OE lines exhibited obvious silique development defects, including shorter siliques and bent siliques (Figure 7). Further dissection revealed that the seed setting rate varied considerably and was significantly lower in *GmBPC8*-OE lines than in WT.

To investigate the potential mechanism by which *GmBPC8* affects plant seed set, we examined the floral developmental process in transgenic plants. *GmBPC8*-overexpressing lines exhibited pronounced floral developmental defects starting from stage 12 (petals level with long stamen) [35,36], including petals that failed to open or opened incompletely, as well as stamens, pistils, and petals that did not elongate properly, ultimately becoming twisted or bent (Figure 8 and Figure S6). These results indicate that *GmBPC8* is involved in the regulation of proper flower opening.

### 2.8. Analysis of Interaction Between GmBPC Proteins

Previous studies have shown that BPC proteins can form homodimers or heterodimers [10,14,37]. The CDS of *GmBPC8* was cloned into pGBT7 (BD), and the CDSs of *GmBPC1*, *GmBPC6*, *GmBPC8*, and *GmBPC9* were cloned into pGADT7 (AD). Different combinations were co-transformed into Y2H Gold yeast cells. We found that GmBPC8 protein had no autonomous activation (Figure 9). Furthermore, the Y2H assay showed that GmBPC8 could interact with itself to form homomers. As shown in Figure 9, GmBPC8 could interact with GmBPC1 and GmBPC6 to form heterodimers. GmBPC8 and GmBPC9 show weak interaction. Collectively, GmBPC8 protein can form both homodimers and heterodimers.

## 3. Discussion

BPC proteins widely exist in plants, and are involved in many aspects of plant development andstress responses. In recent years, the *BPC* gene family has been reported in multiple plant species. However, few studies have been reported on BPC family in soybean. Here, we identified ten *GmBPC* genes in the soybean genome using BLASTP and HMM search (Table 1). Subsequently, we carried out a systematic analysis of the *GmBPC* family, including phylogenetic relationships, chromosome locations, conserved domains, and *cis*-regulatory elements. Based on the phylogenetic analysis, *GmBPCs* were classified into two groups: 4 *GmBPC* genes in group I and 6 *GmBPC* genes in group II (Figure 1), consistent with the classification reported in *Arabidopsis thaliana*, apple, cucumber, and tomato [14,29,32]. Combining conserved motif and domain analysis, all GmBPC proteins contain five conserved cysteine residues in the C-terminus and have differences in the N-terminus (Figure 3 and Figure S2). Previous studies have revealed that Cysteine residues play indispensable roles in homodimerization and protein structural stability through disulfide bonds [34]. The conserved amino acid in the C-terminus of *Arabidopsis AtBPC1* is vital for recognizing GAGA-sequences [30]. *GmBPCs* within the same group had similar motif compositions and exon/intron patterns, indicating a closer evolutionary relationship (Figure 3). Therefore, these data support the classification and imply that *GmBPCs* within the same group may share similar functional characteristics.

Tissue-specific expression levels of genes are commonly associated with their functions [38]. Thus, we carried out expression profile analysis of *GmBPC* genes in nine different soybean tissues. We found that most *GmBPCs* were expressed in all tissues. Some *GmBPCs* exhibited tissue-specific expression, indicating that they may have distinct functions during plant development (Figure 5). The expression profiles of BPC genes varied among different plant species [29,31,32]. For instance, cucumber *CsBPC* genes showed differential expression in different tissues, with all *CsBPCs* highly expressed in seeds [29]. Several studies have displayed that BPC genes respond to abiotic stresses [9]. All *GmBPCs* were induced by drought stress. Most *GmBPCs* were prominently upregulated under heat stress, although the fold change was relatively small (Figure 6). Similarly, all *CsBPCs* were upregulated in cucumber under drought, salt, heat, and cold stresses [29]. Most tomato *SlBPCs* showed low expression under drought stress, whereas about half were upregulated under salt and heat stresses [32]. These results imply that *GmBPC* genes might play essential roles in soybean abiotic stress responses.

Flower opening is a precisely coordinated biological process that is essential for successful pollination and fertility [36,39,40]. In the present study, overexpression of *GmBPC8* in *Arabidopsis thaliana* caused abnormal petal opening (Figure 8 and Figure S6), which may in turn impede normal pollen dispersal, potentially leading to insufficient pollination and reduced seed setting rate. Similarly, the *Arabidopsis HSP70-16* mutant failed to open flowers due to postgenital fusion at overlapping tips of two lateral sepals, which subsequently affected pollination and seed formation [41]. However, whether *GmBPC8* regulates flower development through similar processes involving organ fusion remains to be further investigated. BPC transcription factors function in reproductive development across species, but with distinct regulatory modes. In *Arabidopsis thaliana*, BPC members show functional redundancy, with the *bpc1-1 bpc2 bpc4 bpc6* quadruple mutant exhibiting reduced fertility and ovule defects, whereas BPC3 acts antagonistically [16]. In *Camellia japonica*, overexpression of *CjBPC1* in *Arabidopsis* affects seed development [13]. We found that overexpression of *GmBPC8* causes floral developmental defects and reduces seed setting rate, which supports a conserved role of BPC proteins in reproductive development. Although overexpression of *GmBPC8* in *Arabidopsis thaliana* resulted in defects in flower opening and seed set, these phenotypes cannot be directly extrapolated to the endogenous function of *GmBPC8* in the native soybean host. Therefore, further validation via stable transformation in soybean is necessary. Nevertheless, the present findings provide important insights into the biological function of *GmBPC8*.

In higher plants, BPC proteins may form complexes with other regulatory factors to activate or repress the expression of target genes [16,37]. BPC4 interacts with PRC2 subunit SWINGER to regulate lateral root development by repressing *ABI4* expression [10]. Similarly, the interaction between MdBPC2 and MdLHP1a/b may be vital for H3K27me3 modification to suppress the expression of *MdYUC2a* and *MdYUC6b* in apple [14]. A weak interaction between BPC1 and BPC6 was found in *Arabidopsis thaliana* by Y2H assay [34]. In this study, GmBPC8 was found to interact with itself to form homodimers. GmBPC8 interacted with GmBPC1 and GmBPC6 (Figure 9). Interactions among GmBPC proteins may alter their binding affinity to downstream target genes, thereby regulating GmBPC8-mediated plant development. These hypotheses require further investigation.

## 4. Materials and Methods

### 4.1. Identification of BPCs in Soybean

To obtain the soybean BPC proteins, the whole-genome sequence and annotation files of soybean (Wm82.a4.v1) were downloaded from the Phytozome database (https://phytozome-next.jgi.doe.gov/, accessed on 4 January 2025). Meanwhile, the protein sequences of seven *Arabidopsis* BPC transcription factors (AtBPC1 to AtBPC7) were retrieved from TAIR (https://www.arabidopsis.org/, accessed on 19 January 2025). These AtBPC sequences were used as queries to search against the soybean genome using BLASTP with E-value threshold of ≤1 × 10^−5^ [32]. Additionally, we utilized the Hidden Markov Model (HMM) profile of the BPC family (PF06217) to perform an HMMER search using TBtools (v2.303). By combining the results of both the BLASTP and HMMER searches, a total of ten candidate GmBPC proteins were ultimately identified in the soybean genome. Candidate proteins were verified using the NCBI-CDD (https://www.ncbi.nlm.nih.gov/cdd/, accessed on 19 January 2025) and SMART (https://smart.embl-heidelberg.de/, accessed on 19 January 2025). The ten *GmBPC* genes were named *GmBPC1* to *GmBPC10* in ascending order according to their physical positions on soybean chromosomes. The physicochemical properties of GmBPC proteins, including molecular weight (kDa), theoretical isoelectric point (pI), and grand average of hydropathicity (GRAVY), were predicted using the ExPASy ProtParam tool (https://web.expasy.org/protparam/, accessed on 20 January 2025).

### 4.2. Sequence Alignment, Conserved Motif, Gene Structure, and Phylogenetic Analysis

The protein sequences of GmBPCs were aligned using DNAMAN 6.0 software. The conserved motifs were identified using MEME (https://meme-suite.org/meme/, accessed on 22 January 2025). The number of motifs was set to 10. The exon–intron structures of the *GmBPC* genes were determined by comparing their coding sequences with the corresponding genomic sequences retrieved from the soybean genome. The gene structures were visualized using TBtools to illustrate the distribution of untranslated regions (UTRs), exons, and introns [42]. The phylogenetic tree of BPC protein sequences was constructed using MEGA 5 software with the neighbor-joining (NJ) method and 1000 bootstrap replicates [43]. Next, the phylogenetic tree was modified using the iTOL V7 (https://itol.embl.de/, accessed on 13 September 2026).

### 4.3. Chromosome Location, Synteny Analysis, and Cis-Regulatory Elements of GmBPCs

Based on the genome annotation of *Glycine max*, the chromosomal locations of *GmBPC* genes were obtained and visualized using TBtools. The synteny analysis of *GmBPC* genes was carried out using MCScanX of TBtools to identify segmental duplication events [5]. The syntenic relationships among *GmBPC* genes were visualized using the Advanced Circos module implemented in TBtools. The 2000 bp promoter sequences upstream of the start codons of *GmBPC* genes were extracted from the soybean genome. These promoter sequences were submitted to the PlantCARE database (https://bioinformatics.psb.ugent.be/webtools/plantcare/html/, accessed on 24 January 2025) for *cis*-acting element prediction. The distribution of these elements was analyzed and visualized using TBtools.

### 4.4. Tissue Expression Analysis of GmBPCs

Transcriptome data of *GmBPCs* in various soybean tissues, including roots, root hairs, nodules, stems, leaves, SAM, pods, seeds, and flowers, were obtained from the Phytozome database (https://phytozome-next.jgi.doe.gov/report/gene/Gmax_Wm82_a2_v1/Glyma.02G182800, accessed on 26 January 2025). The expression values, presented as FPKM, were extracted from RNA-seq datasets. The raw FPKM values were log_2_-transformed and then visualized as heatmaps using TBtools [44,45].

### 4.5. Plant Materials and Stress Treatments

Soybean (Williams 82) seeds were germinated on moistened paper. Uniformly germinated seedlings were then transferred to half-strength Hoagland’s nutrient solution (pH 5.8) and cultivated in a growth chamber under 16/8 h (light/dark) photoperiod at 25 °C. The nutrient solution was renewed every 3 days. At the V1 stage (first trifoliolate leaves), the seedlings were subjected to drought stress and heat stress [44]. For drought stress, the seedlings were treated with 20% PEG6000 solution for 0, 6, 12, and 24 h at 25 °C. For heat stress treatment, the seedlings were transferred to a plant growth incubator set at 42 °C for 0, 6, 12, and 24 h. For RNA extraction, the three leaflets of the first trifoliolate leaves were collected at each time point. For each biological replicate, leaf tissues from three independent plants were pooled together to form a single sample. The samples were immediately frozen in liquid nitrogen and subsequently stored at −80 °C.

### 4.6. Plasmid Construction and Genetic Transformation

The full-length CDS of *GmBPC8* was amplified by PCR using 2 × Phanta^®^ Max Master Mix (Vazyme, Nanjing, China). The amplified fragment was cloned into the expression vector pRed1305 as previously described [46]. The resulting recombinant vector was then introduced into *Agrobacterium tumefaciens* strain GV3101, which was subsequently used to transform *Arabidopsis thaliana* (L.) Heynh. (Col-0) via the floral dip method [47,48]. To identify transgenic plants, genomic DNA was extracted from *Arabidopsis thaliana* leaves using Plant Genomic DNA Kit (TIANGEN, Beijing, China) according to the manufacturer’s instructions, and the *GmBPC8* sequence was detected by PCR using specific primers. All phenotypic analyses were performed using these homozygous T3 lines (OE1–OE3). All primers used are listed in Supplementary Table S1.

### 4.7. RNA Extraction and qRT-PCR Assays

Total RNAs were isolated using the RNA Plant Plus Reagent (Tiangen, Beijing, China) according to the manufacturer’s instructions. RNA was reverse-transcribed into cDNA using PrimScript™ First Strand cDNA Synthesis Kit (TaKaRa, Dalian, China). qRT-PCR was conducted using the CFX96 Real-Time System (Bio-Rad, Hercules, CA, USA) using ChamQ Blue Universal SYBR qPCR Master Mix (Vazyme, Nanjing, China) [49]. The soybean *GmTub* (Glyma.05G157300) was selected as an internal reference [45]. The relative expression levels of *GmBPCs* were calculated based on the 2^−ΔΔCt^ method [50]. Three independent biological replicates were performed for each sample, and each biological replicate contained three technical replicates [51]. The gene primers are listed in Supplementary Table S1.

### 4.8. Y2H Assays

Y2H assays were conducted as previously described [52]. The full-length CDS of *GmBPC8* was cloned into pGBT7, whereas those of *GmBPC1*, *GmBPC6*, *GmBPC8*, and *GmBPC9* were cloned into pGADT7. The bait construct GmBPC8-BD was co-transformed with the prey constructs GmBPC1-AD, GmBPC6-AD, GmBPC8-AD, or GmBPC9-AD, respectively, into the Y2H Gold yeast strain (Clontech) using the polyethylene glycol/lithium acetate (PEG/LiAc) method. The yeast strains were grown on SD/-Trp-Leu (SD/-T/-L) or SD/-Trp-Leu-His-Ade (SD/-T/-L/-H/-A) medium at 30 °C for 3–4 days. The empty pGADT7 vector was used as a negative control. The primers used are listed in Supplementary Table S1.

## 5. Conclusions

In summary, this study presents a genome-wide identification and systematic characterization of the BBR/BPC transcription factor family in soybean. We systematically characterized *GmBPCs*, including their chromosomal distribution, phylogenetic relationships, conserved motifs, gene structures, expression profiles, and protein–protein interactions. We investigated the function of *GmBPC8* in floral organ development and seed set in *Arabidopsis thaliana*. These findings provide insights into *GmBPC* functions in soybean development and lay a foundation for future studies of GmBPC8-mediated downstream networks.

## Figures and Tables

**Figure 1:**
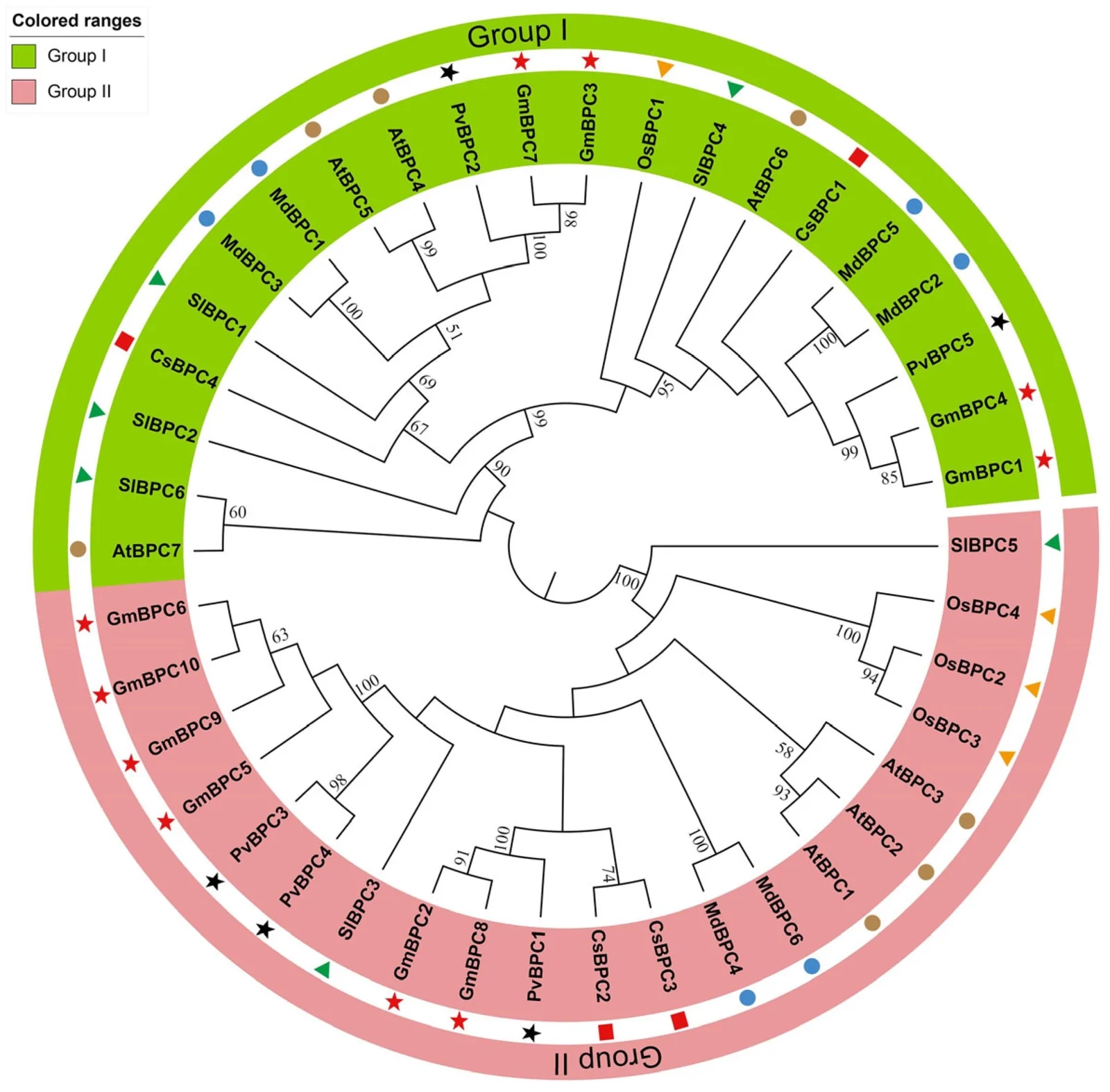
Phylogenetic analysis of BPC proteins. Phylogenetic analysis was performed using BPC protein sequences from seven plant species including *Arabidopsis thaliana*, *Cucumis sativus*, *Glycine max*, *Malus domestica*, *Oryza sativa*, *Phaseolus vulgaris*, and *Solanum lycopersicum*. The phylogenetic tree was constructed using the neighbor-joining method with 1000 bootstrap replicates. The BPC proteins were divided into two major groups (Group I and Group II), indicated by different colored backgrounds.

**Figure 2:**
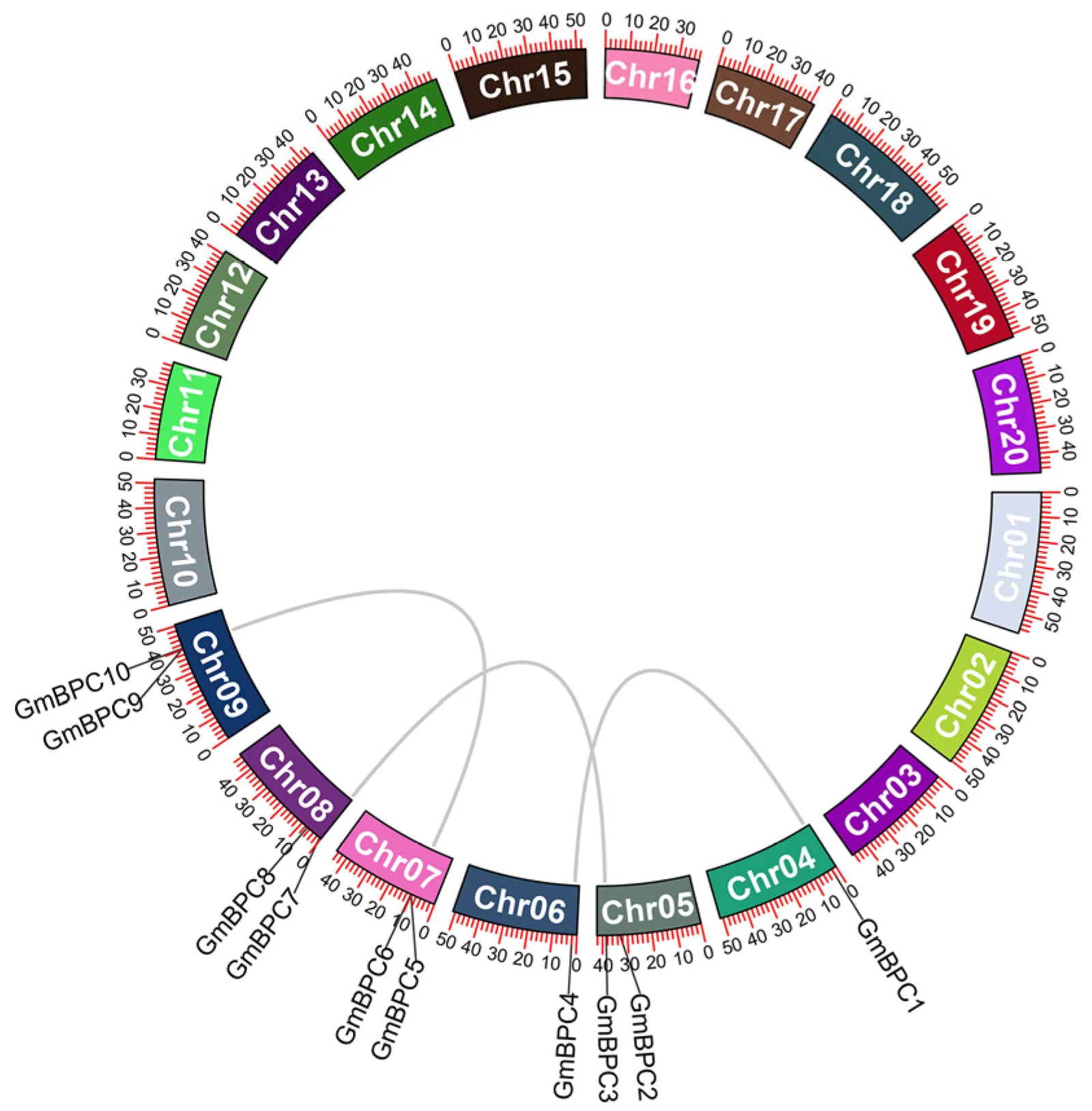
Synteny analysis of *GmBPCs* in the soybean genome. The gray lines represent the duplicated gene pairs of the *GmBPC* genes within the genomes. Three segmental duplication pairs were identified: *GmBPC1*/*GmBPC4*, *GmBPC3*/*GmBPC7*, and *GmBPC5*/*GmBPC9*.

**Figure 3:**
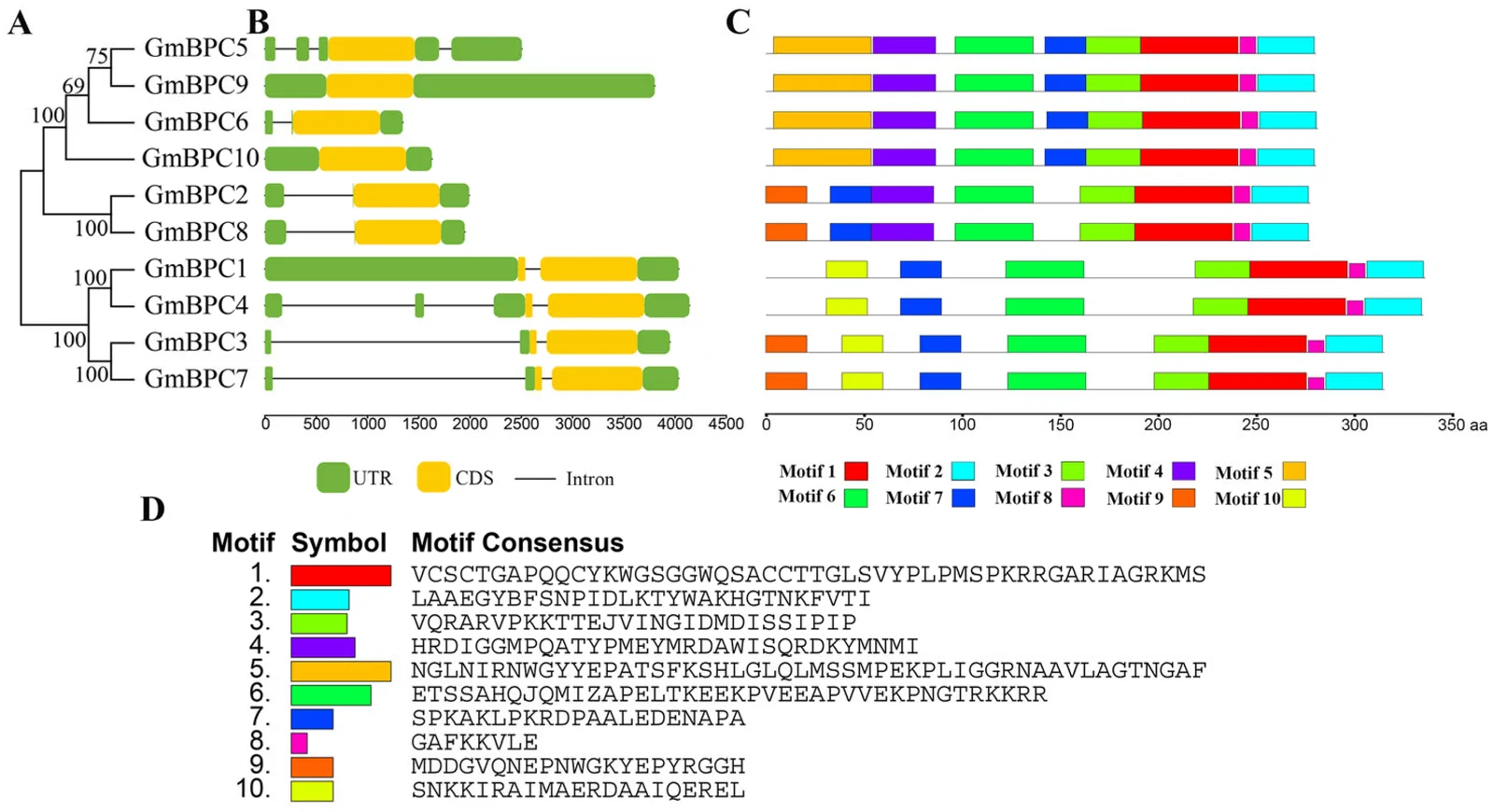
The gene structure and motif analysis of *GmBPCs*. (**A**) The evolutionary tree of GmBPC proteins. (**B**) The gene structure of *GmBPCs* shows the exon–intron organization. Yellow boxes represent exons (CDS), and black lines represent introns. (**C**) The motifs of GmBPC proteins. Ten conserved motifs (motif 1 to motif 10) were identified using the MEME tool, with different colored boxes representing each motif. (**D**) The motif sequence of GmBPC proteins.

**Figure 4:**
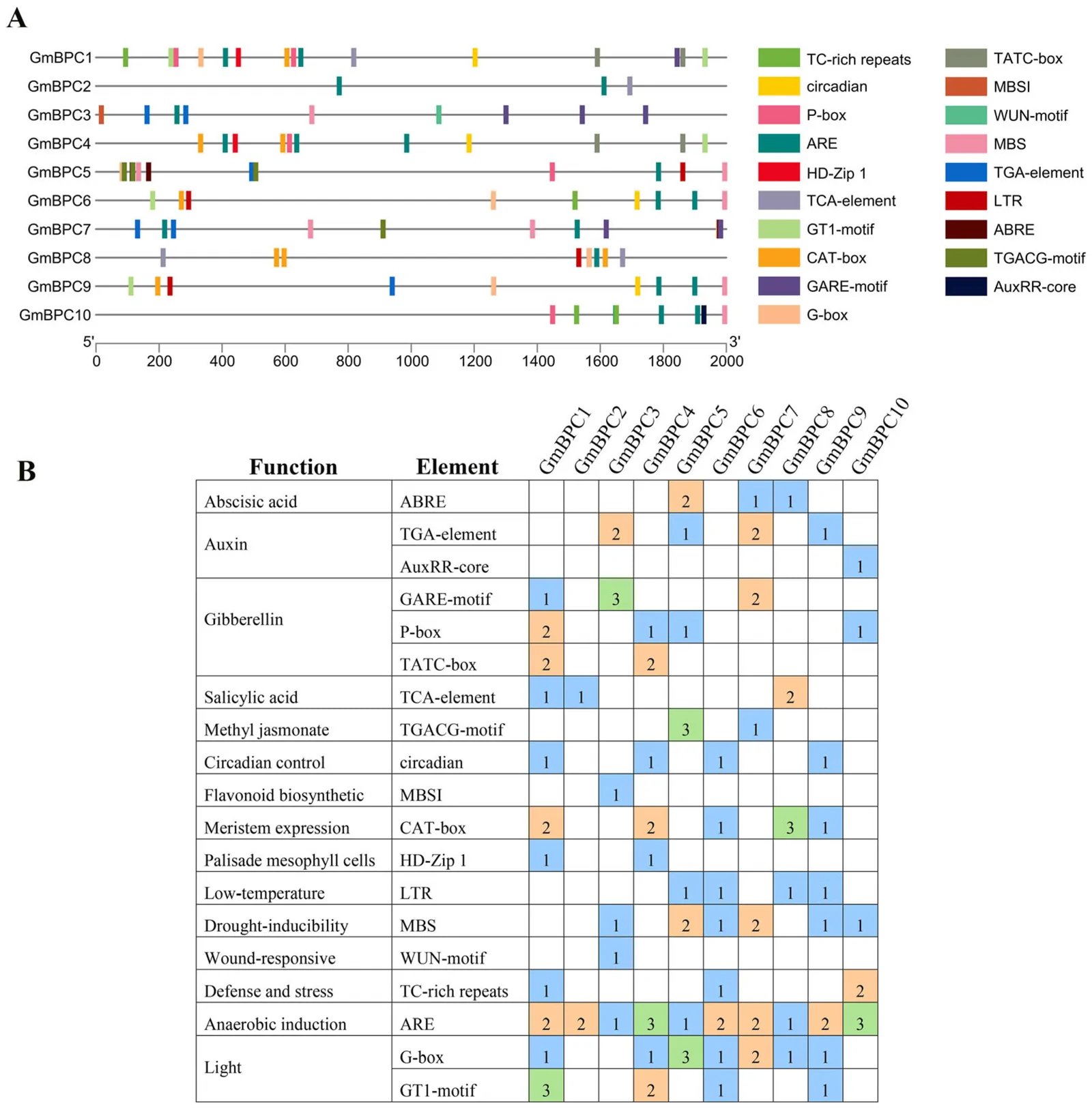
*Cis*-acting elements analysis of *GmBPCs*. (**A**) Distribution of *cis*-acting elements in the promoter regions (2000 bp upstream of the start codon) of *GmBPC* genes. The *cis*-acting elements were identified using the PlantCARE database. Different colored boxes represent distinct types of *cis*-elements. (**B**) The statistical information of *cis*-acting elements in *GmBPC* promoters.

**Figure 5:**
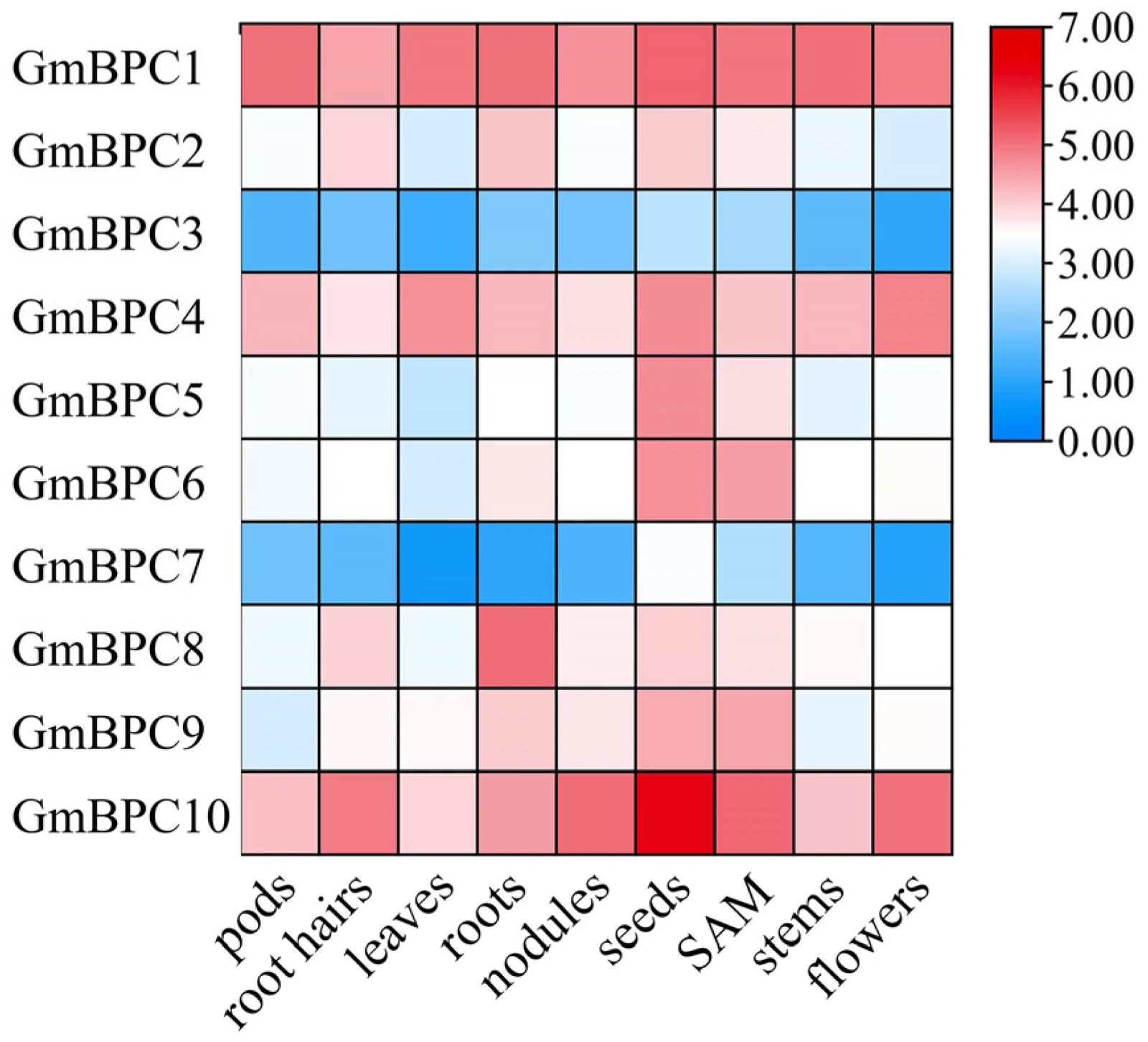
Expression profiles of *GmBPCs* in various tissues. Heat map showing the transcript levels of *GmBPC* genes across nine tissues: roots, root hairs, nodules, stems, leaves, SAM, pods, seeds, and flowers. The expression data were obtained from the Phytozome database and normalized for visualization. The heat map was generated by TBtools (v2.303). The color scale from blue to red represents relative expression levels.

**Figure 6:**
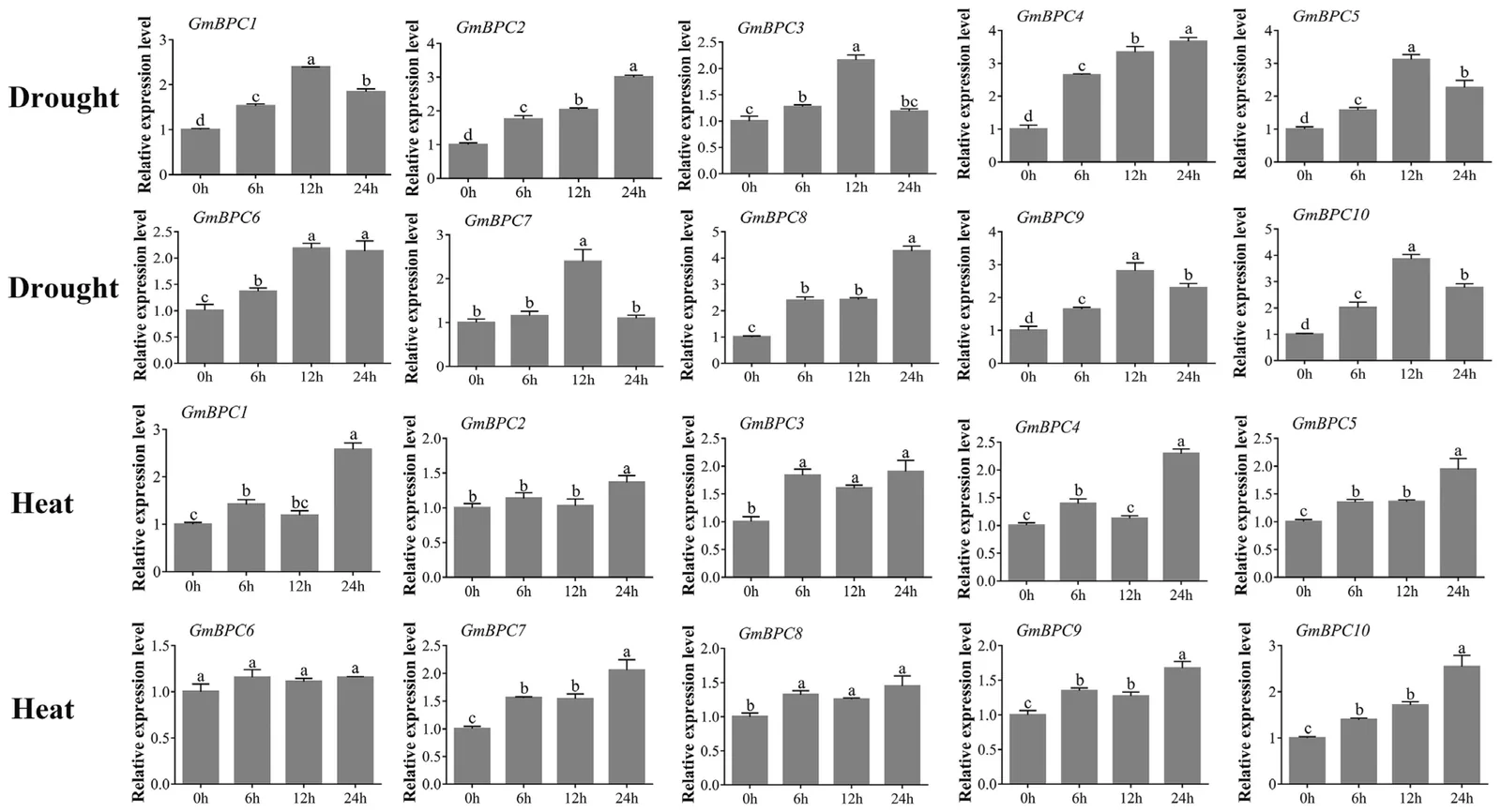
Expression patterns of *GmBPCs* in different stresses. Soybean seedlings were exposed to 20% PEG 6000 and heat (42 °C) conditions. The expression levels of *GmBPCs* were detected by qRT-PCR. Data are presented as the mean ± SD from three independent biological replicates, each with three technical replicates. Different letters indicate significant differences (*p* < 0.05, one way ANOVA with Tukey’s test).

**Figure 7:**
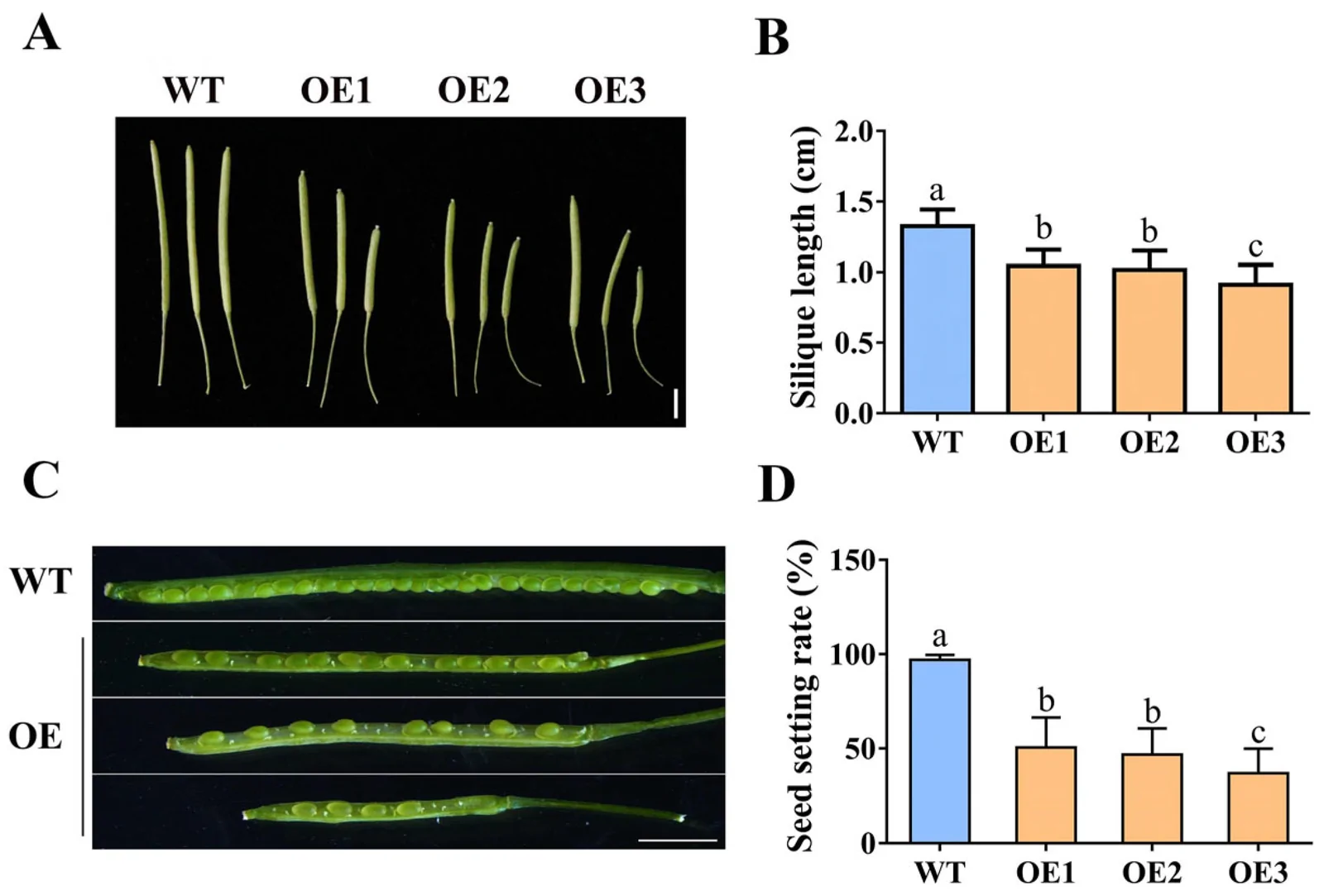
Overexpression of *GmBPC8* affects silique development and seed setting rate. (**A**) Silique phenotypes of WT and *GmBPC8*-OE lines. (**B**) Silique length among WT and OE lines. (**C**) Open siliques of WT and OE lines. Scale bars = 2 mm. (**D**) Seed setting rate of WT and *GmBPC8*-OE lines. Values represent the mean ± SD of three biological replicates (*n* = 30 siliques). Different lowercase letters indicate significant differences (*p* < 0.05, one way ANOVA with Tukey’s test).

**Figure 8:**
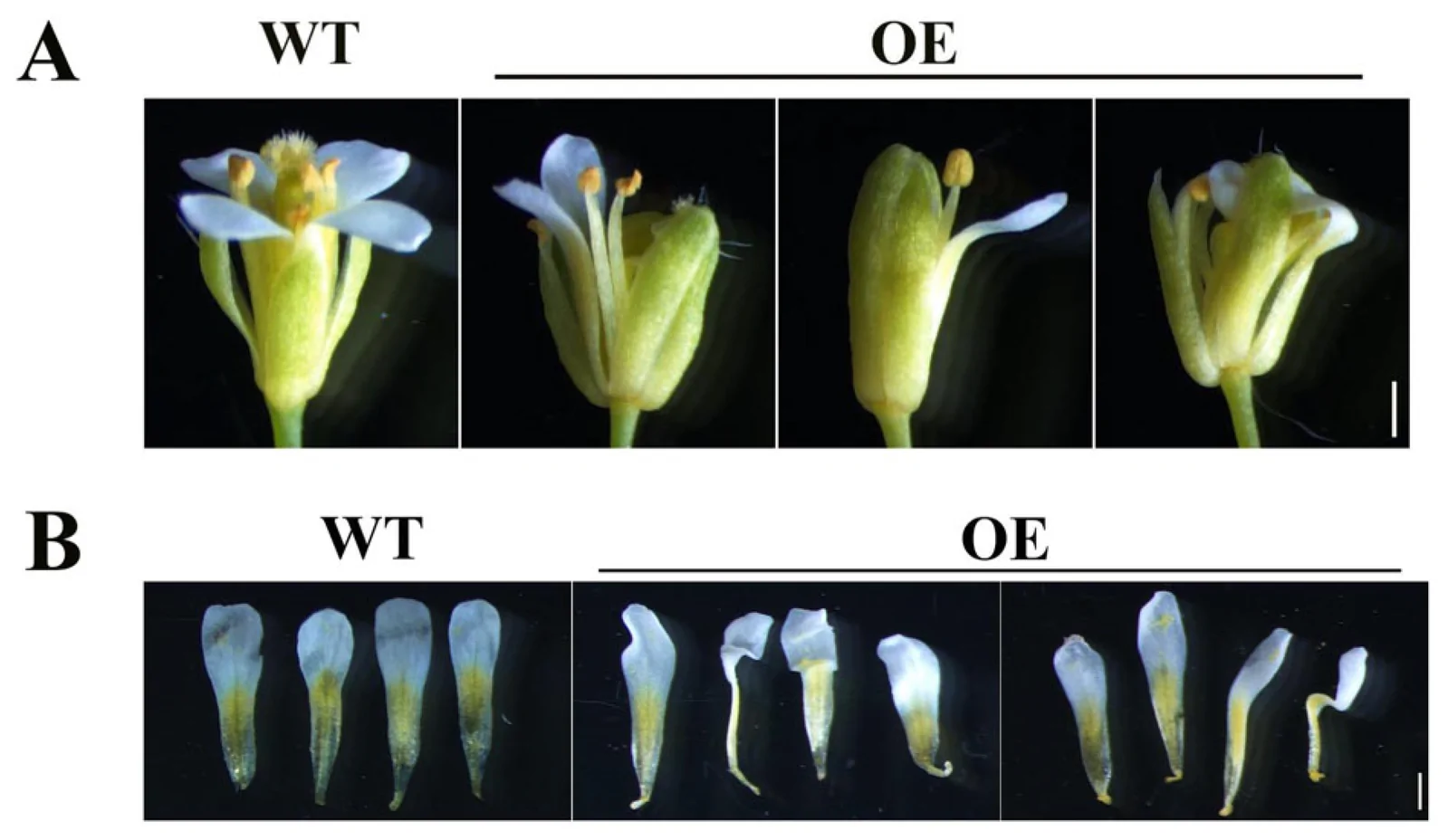
Overexpression of *GmBPC8* affects floral development. (**A**) Floral developmental status of WT and *GmBPC8*-OE lines at flower stage 14 (long anthers extend above stigma). Scale bars = 0.5 mm. (**B**) Petal phenotypes of WT and OE lines. At least 30 flowers from 5 plants were observed across three biological replicates. Scale bars = 0.5 mm.

**Figure 9:**
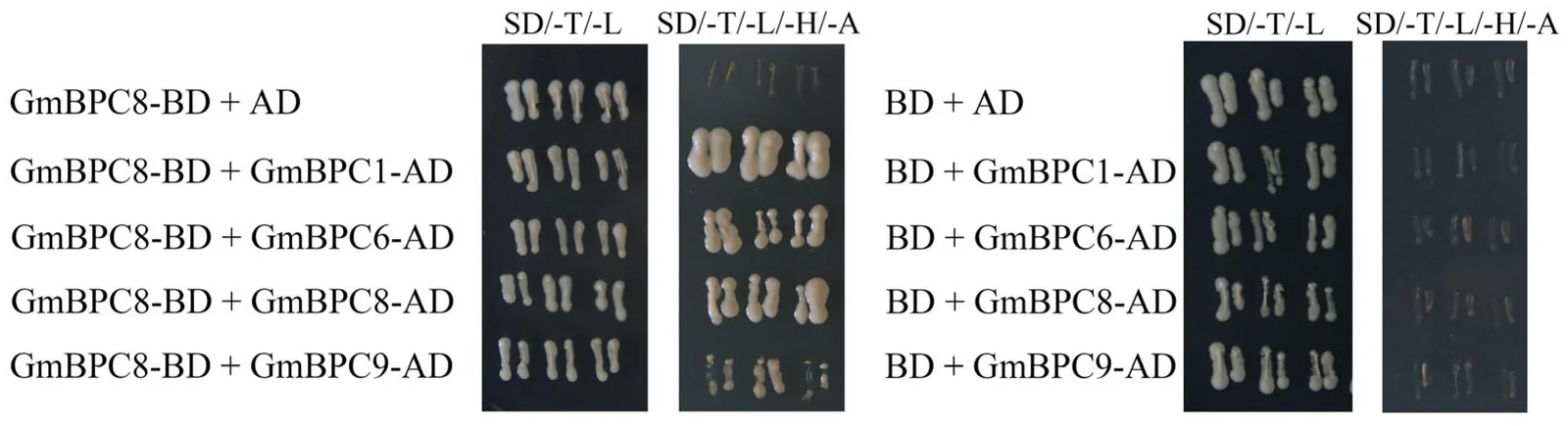
Y2H assay testing the interactions between GmBPC8 and GmBPC proteins. Different combinations of the *GmBPC8*-BD construct (or empty BD vector) with the empty AD vector, GmBPC1-AD, GmBPC6-AD, GmBPC8-AD, or GmBPC9-AD were co-transformed into Y2H Gold yeast cells. Transformants were grown on SD/–Leu/–Trp and SD/–Leu/–Trp/–His/–Ade media at 30 °C.

**Table 1:** Summary of the *GmBPC* genes.

Gene Locus	Gene Name	Gene Length (bp)	Number of Amino Acids	Molecular Weight	Theoretical pI	GRAVY
Glyma.04G026300	GmBPC1	4036	338	37,449.46	9.47	−0.838
Glyma.05G137100	GmBPC2	1993	279	30,943.53	9.54	−0.683
Glyma.05G201900	GmBPC3	3950	317	35,583.33	9.35	−0.687
Glyma.06G026100	GmBPC4	4141	337	37,455.40	9.42	−0.874
Glyma.07G107300	GmBPC5	2507	282	31,293.13	9.88	−0.638
Glyma.07G107400	GmBPC6	1345	283	31,246.97	9.73	−0.648
Glyma.08G009300	GmBPC7	4035	317	35,553.34	9.35	−0.679
Glyma.08G092400	GmBPC8	1950	279	31,348.23	9.63	−0.666
Glyma.09G170300	GmBPC9	3802	282	31,228.96	9.77	−0.645
Glyma.09G170400	GmBPC10	1629	282	31,234.94	9.75	−0.664

## Data Availability

The transcriptome data of soybean various tissues were obtained from the Phytozome database (https://phytozome-next.jgi.doe.gov/report/gene/Gmax_Wm82_a2_v1/Glyma.02G182800, accessed on 26 January 2025).

## Supplementary Materials

The following supporting information can be downloaded at: https://www.mdpi.com/article/10.3390/plants15192940/s1, Figure S1. Chromosomal distribution of ten *GmBPC* genes; Figure S2. Conserved domain of GmBPC proteins. Red lines represent the DNA-binding domain of GmBPC proteins; Figure S3. The transgenic *Arabidopsis* lines were identified by PCR; Figure S4. The transgenic *Arabidopsis* lines were identified by qRT-PCR. Different letters indicate significant differences (*p* < 0.05, one way ANOVA with Tukey’s test); Figure S5. Plant height of WT and transgenic *Arabidopsis* lines. Different letters indicate significant differences (*p* < 0.05, one way ANOVA with Tukey’s test); Figure S6. Observation of abnormal pistil bending in transgenic *Arabidopsis*. Scale bars = 0.5 mm; Table S1. Primers used in this study.
