# Supplementary material for: Genome-Wide Analysis of BPC Gene Family in Soybean and the Role of GmBPC8 in Floral Organ Development in Arabidopsis
Source: Plants (Basel). 2026 Sep 26;15(19):2940. doi: 10.3390/plants15192940 (PMC13666826; doi:10.3390/plants15192940)
Supplement: Supplementary file 1 [file plants-15-02940-s001.zip › plants-4519130-supplementary.pdf]

Supplementary data

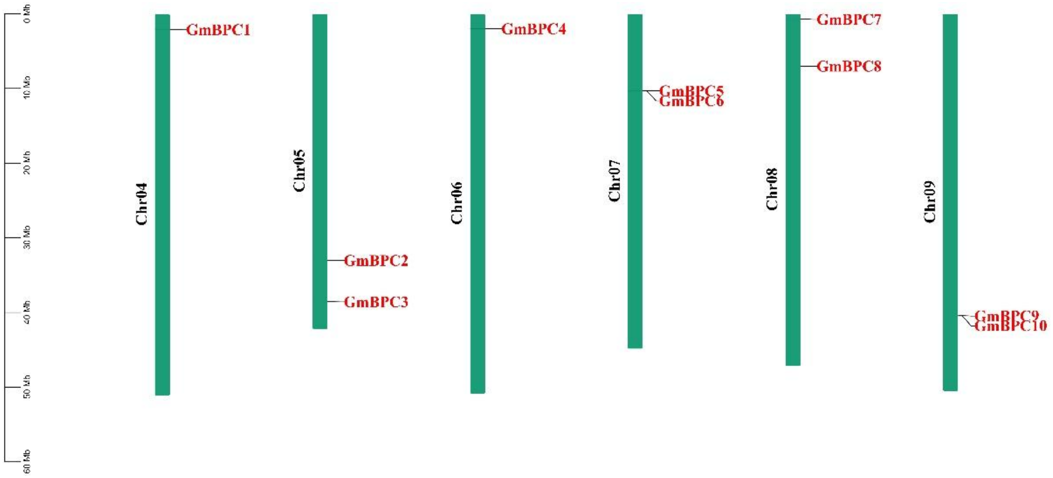

Figure S1. Chromosomal distribution of ten *GmBPC* genes.

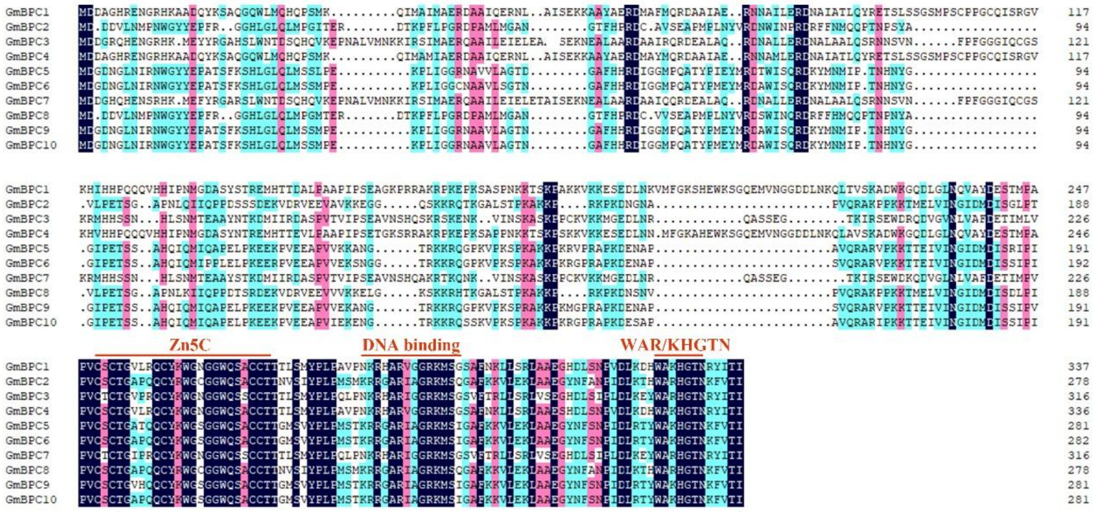

Figure S2. Conserved domain of GmBPC proteins. Red lines represent the DNA-binding domain of GmBPC proteins.

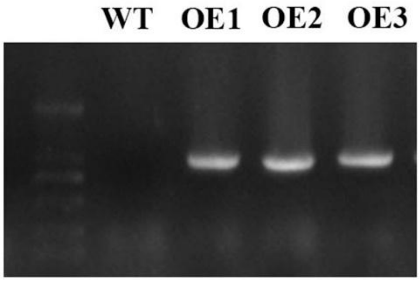

Figure S3. The transgenic *Arabidopsis* lines were identified by PCR.

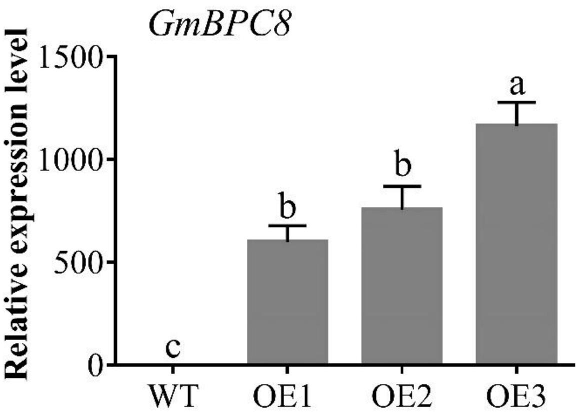

Figure S4. The transgenic *Arabidopsis* lines were identified by qRT-PCR. Different letters indicate significant differences (P < 0.05, one way ANOVA with Tukey's test).

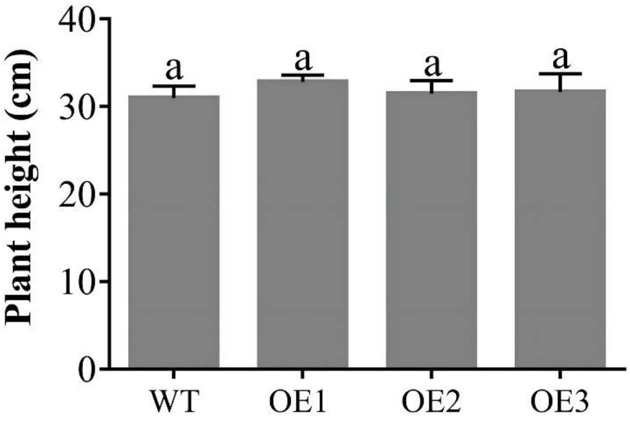

Figure S5. Plant height of WT and transgenic *Arabidops*is lines. Different letters indicate significant differences (P < 0.05, one way ANOVA with Tukey's test).

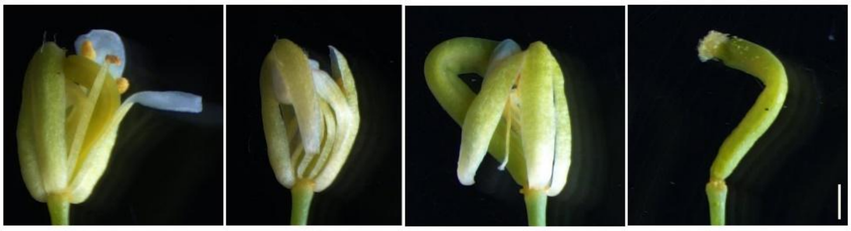

Figure S6. Observation of abnormal pistil bending in transgenic *Arabidopsis*. Scale bars=0.5 mm.

Table S1. Primers used in this study.

Primer name	sequence (5' to 3')
MdBPC1(qRT)-F	GTATGCCATCATGCCCTCCA
MdBPC1(qRT)-R	GGCATCGGTTGTGTGCATTT
MdBPC2(qRT)-F	CGGTTTGTTCATGCACTGGG

MdBPC2(qRT)-R	TTGCACCACGCCTTTTCATG
MdBPC3(qRT)-F	GGCAGAGGGATGAAGCACTT
MdBPC3(qRT)-R	TGCATCCGTTTCGATCCACA
MdBPC4(qRT)-F	TGCCCAACAAGAGACATGCT
MdBPC4(qRT)-R	GGTCCTTGAGGTCAACAGGG
MdBPC5(qRT)-F	CAAGCACCGGAATTGCCAAA
MdBPC5(qRT)-R	GGAACCCTTGCACGTTGAAC
MdBPC6(qRT)-F	GTTCAACGTGCAAGGGTTCC
MdBPC6(qRT)-R	ACATCCCTGTCGTGCAACAT
MdBPC7(qRT)-F	ATAATGCCCTTGCTGCCCTT
MdBPC7(qRT)-R	ACCGGGGAGGCATCTCTTAT
MdBPC8(qRT)-F	TTGTTCGTGTACTGGGGCTC
MdBPC8(qRT)-R	TTGCACCACGCCTTTTCATG
MdBPC9(qRT)-F	TGGAAGAAGCACCTGTCGTC
MdBPC9(qRT)-R	GGAACCCTTGCACGTTGAAC
MdBPC10(qRT)-F	TCAACGTGCAAGGATTCCGA
MdBPC10(qRT)-R	CAGACTGCCAACCACCAGAT
β-tubulin-F	GGAAGGCTTTCTTGCATTGGTA
β-tubulin-R	AGTGGCATCCTGGTACTGC
AtActin-F	TTTGGAGCCTGGGACTATGGAT
AtActin-R	ACGGGGGAATGGGATGAGAT
GmBPC1-AD-F	GAGGCCAGTGAATTCATGGATGATGCCGGGCAT
GmBPC1-AD-R	GAGCTCGATGGATCCCTTTATTGTGATGTAACGGTTCG
GmBPC6-AD-F	GAGGCCAGTGAATTCATGGATGGTGATAATGGTC
GmBPC6-AD-R	GAGCTCGATGGATCCCCTAATAGTGACAAACTTGTTG
GmBPC8-AD-F	GAGGCCAGTGAATTCATGGATGATGATGTGTTG
GmBPC8-AD-R	GAGCTCGATGGATCCCCTGATAGTGACAAACTT
GmBPC9-AD-F	GAGGCCAGTGAATTCATGGATGGTGATAATGGTC
GmBPC9-AD-R	GAGCTCGATGGATCCCCTAATGGTGACAAACTTG
GmBPC8-BD-F	ATGGAGGCCGAATTCATGGATGATGATGTGTTG
GmBPC8-BD-R	CAGGTCGACGGATCCCCTGATAGTGACAAACTT
GmBPC8-F	CTCGGTACCCGGGGATCCATGGATGATGATGTGTTG
GmBPC8-R	GCATGCCTGCAGGTCGACCTACCTGATAGTGACAAACTTG
